# Dual-Platform Mushroom Cultivation for STEM Education: AI-Assisted Environmental Monitoring and Student Perceptions

**DOI:** 10.3390/educsci16071010

**Published:** 2026-06-26

**Authors:** Byron Meade, Annie Wang, Steven Layne, Emily Duncan, Brooke Duncan, Eli Johnson, Lucas Gibson, Teresa Johnson, Ivan Wheeling, Grant Lumpkins, Daniel Flores, Walden Martin, Kevin Wang

**Affiliations:** Division of Math and Natural Sciences, University of Pikeville, Pikeville, KY 41501, USA

**Keywords:** artificial intelligence, controlled-environment agriculture, mushroom cultivation, experiential STEM education, student perception

## Abstract

A dual-platform mushroom cultivation system integrating artificial intelligence (AI)-assisted environmental monitoring and controlled-environment agriculture (CEA) was developed to support experiential STEM education across K–12 and undergraduate settings. Hands-on instruction with multicellular fungi is often limited by reliance on microbial models and by constraints associated with field-based activities. To address this gap, we implemented an indoor instructional platform that combines a commercial AI-assisted automated cultivation unit with a tent-based chamber for hands-on environmental control. Representative cultivated species included oyster mushrooms (*Pleurotus* spp.) and lion’s mane (*Hericium erinaceus*). The AI-assisted system provided sensor/camera-based monitoring, app-based feedback, and software-assisted regulation of humidity, light, and airflow, whereas the tent-based system enabled direct student manipulation of cultivation conditions. Together, the systems allowed students to observe fungal development, manage environmental parameters, and collect quantitative and qualitative data within a single academic term. Post-harvest activities, including mushroom-based food preparation and tasting, further connected fungal biology with food and sustainability. A matched pre- and post-course survey (*n* = 30) showed increases in students’ self-reported perceived understanding, cultivation confidence, and engagement, with mean scores increasing from approximately 2–4 to 6–8. Because the survey instrument was not formally validated and no control group was included, these results are interpreted as preliminary self-reported perceptions rather than objective evidence of learning gains. The platform provides a practical model for integrating fungal biology, AI-assisted environmental monitoring, and CEA into STEM education.

## 1. Introduction

Fungi play fundamental roles in ecosystem function, nutrient cycling, agriculture, biotechnology, and medicine (Amara & El-Baky, 2023; Wang et al., 2025; Nygård & Meyer, 2026; Schanz & Remmele, 2026). Despite their importance, hands-on instruction involving multicellular fungi remains limited in both K–12 and undergraduate education (Choong, 2022; Hardy, 2026). Although fungal biology is introduced across General Biology, Microbiology, Ecology, and Biotechnology courses, the specific instructional gap addressed in this study is the limited availability of hands-on, macrofungi-based laboratory systems that are practical for semester-long STEM instruction. Laboratory exercises commonly use tractable microbial models, including bacterial or yeast, because they are easier to grow, develop rapidly, and require less specialized infrastructure. By contrast, macrofungal cultivation requires coordinated environmental conditions, including humidity, airflow, light, and substrate management (Rämä & Quandt, 2021; Jayaraman et al., 2024; Jacob et al., 2025), as well as sufficient time for mycelial expansion and fruiting-body development (Marshall, 2018; Delvendahl et al., 2022; Dos Reis et al., 2024; Lai et al., 2025). Consequently, students may have limited opportunities to cultivate macrofungi directly, observe visible developmental transitions, and connect fungal growth with controlled-environment agriculture, automation, and quantitative data collection.

Traditional instruction involving mushrooms often relies on field-based activities in which students observe or collect wild species (Santiago et al., 2016; Kratz et al., 2024). While these approaches provide valuable ecological context, they are constrained by seasonality, environmental variability, and safety concerns related to species identification and toxicity (Cazabonne et al., 2022; Karakaya et al., 2022). These limitations make consistent integration of macrofungal study into laboratory-based instruction challenging, particularly at primarily undergraduate institutions (PUIs) and in rural regions such as Central Appalachia, where access to controlled-environment agricultural infrastructure is limited (Hewlett, 2018; Alvares et al., 2022; Sowl et al., 2022).

At the same time, advances in artificial intelligence (AI), sensors, and automated environmental control systems provide new opportunities to support data-driven, controlled-environment learning in biology education (Johnson et al., 2024; Nagarsheth et al., 2025; Omotayo et al., 2025). To address these challenges, we developed an indoor mushroom cultivation platform integrating two complementary systems: an AI-assisted automated unit and a tent-based cultivation chamber for hands-on environmental manipulation. The AI-assisted system supports real-time monitoring and software-assisted regulation of growth conditions, promoting reproducibility and data-centered learning (Aijaz et al., 2025). In contrast, the tent-based system emphasizes direct student interaction, experimental design, and system-level understanding. Together, these systems form a flexible instructional framework adaptable across educational levels, from K–12 outreach to advanced undergraduate course-based undergraduate research experiences (CUREs).

Implemented within an existing laboratory at a rural PUI, this platform allows students to observe fungal developmental processes, manipulate environmental parameters, and collect quantitative growth data within a single academic semester. This study describes the design, implementation, and educational applications of this dual-platform system as a scalable and accessible approach for integrating fungal biology and controlled-environment agriculture (CEA) into STEM education (Boyd et al., 2025).

The implementation is grounded in experiential and inquiry-based STEM education, in which students learn through direct observation, data collection, interpretation, and reflection. By combining automated environmental monitoring with hands-on cultivation, the platform supports emerging data literacy and AI literacy by helping students connect sensor-generated information, environmental conditions, and biological growth responses. The system also provides a foundation for future CURE-based instruction involving student-generated hypotheses, experimental design, and formal assessment.

## 2. Materials and Methods

### 2.1. Educational Setting and Instructional Design

The mushroom cultivation platform was implemented in an undergraduate biology laboratory at a PUI and integrated into course-based instruction, experiential learning activities, and outreach demonstrations. The instructional design combined automated monitoring with hands-on cultivation to support both introductory and advanced learners.

The platform was structured to align with experiential learning and CURE principles by enabling students to observe biological processes, manipulate environmental variables, and interpret growth-related data. It was also adapted for K–12 outreach through laboratory visits and demonstrations.

### 2.2. Mushroom Cultivation Workflow

**Figure 1.**
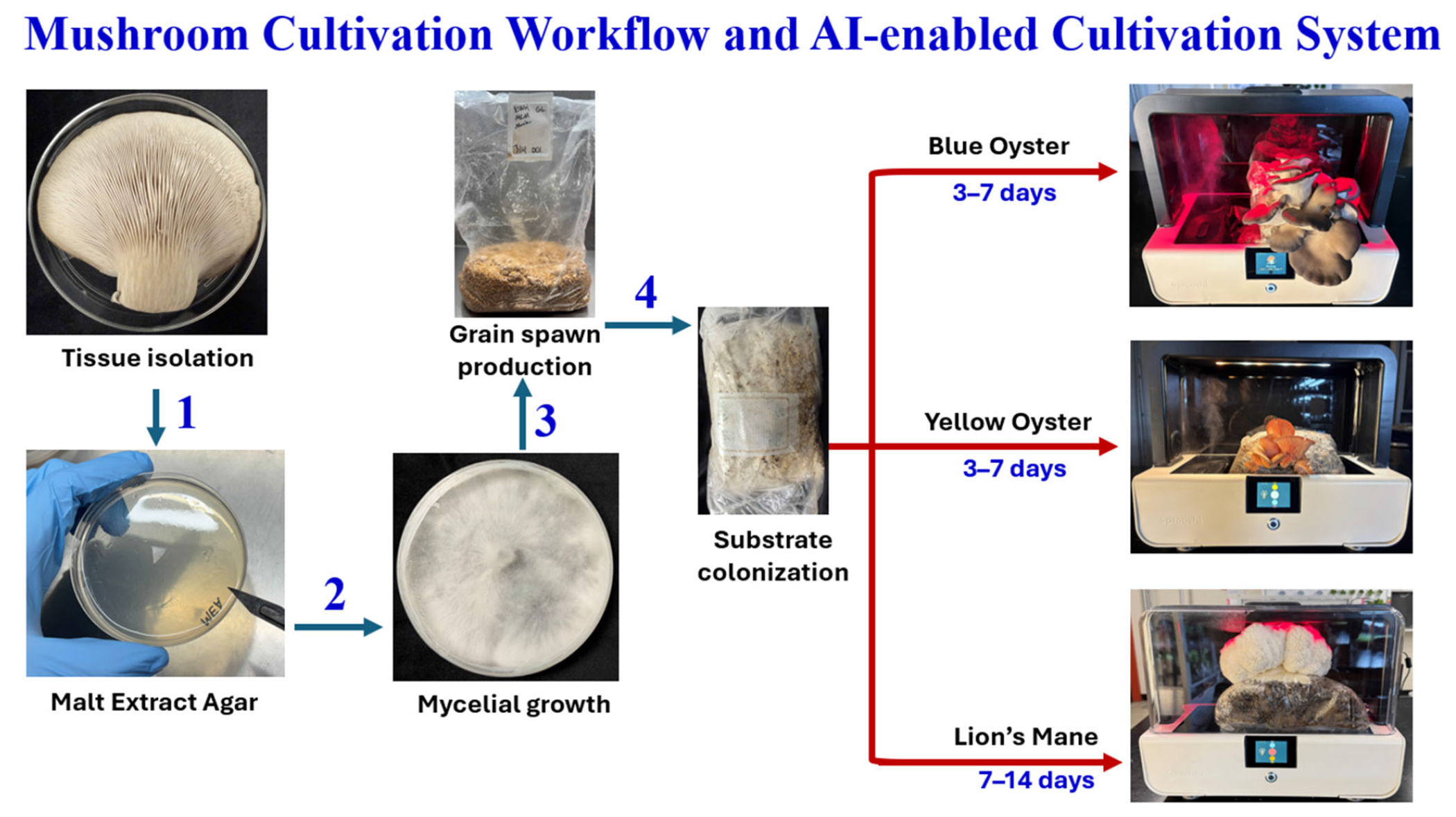
Mushroom cultivation workflow. (1) Tissue isolation from fresh fruiting bodies onto agar plates under aseptic conditions. (2) Mycelial expansion on Malt Extract Agar (MEA) to establish pure cultures. (3) Transfer of agar plugs to sterile grain (millet) for spawn production. (4) Inoculation of bulk substrate and colonization in filter patch bags. Fully colonized substrates were transferred to the AI-assisted cultivation system (Sproushi) for automated environmental control. Representative fruiting of different species, including blue oyster (*Pleurotus* spp.), yellow oyster (*Pleurotus* spp.), and lion’s mane (*Hericium erinaceus*), is shown, with typical fruiting times ranging from 3–14 days depending on species and conditions.

Mushroom cultivation was performed using a standardized four-step workflow (Figure 1), including tissue isolation, mycelial expansion, spawn production, and substrate colonization.

Briefly, inner tissue from fresh fruiting bodies was aseptically excised and cultured on Malt Extract Agar (MEA) to establish pure mycelial cultures. MEA was prepared using 30 g malt extract, 5 g mycological peptone, and 15 g agar per liter.

After 5–10 days of mycelial growth, agar plugs were transferred to sterile grain (millet) for spawn production. Fully colonized grain spawn was subsequently used to inoculate bulk substrate in filter patch bags (Unicorn 3B, 5 μm, Mushroom Media Online, Jefferson City, MO, USA), where substrate colonization proceeded under controlled conditions until full mycelial coverage was achieved.

Incubation was conducted at 21–24 °C in the absence of light. Fruiting was induced by modifying environmental conditions, including exposure to light, increased fresh air exchange, reduced CO_2_ levels (<600 ppm), and elevated relative humidity (80–95%).

### 2.3. Dual-Platform Cultivation System

**Figure 2.**
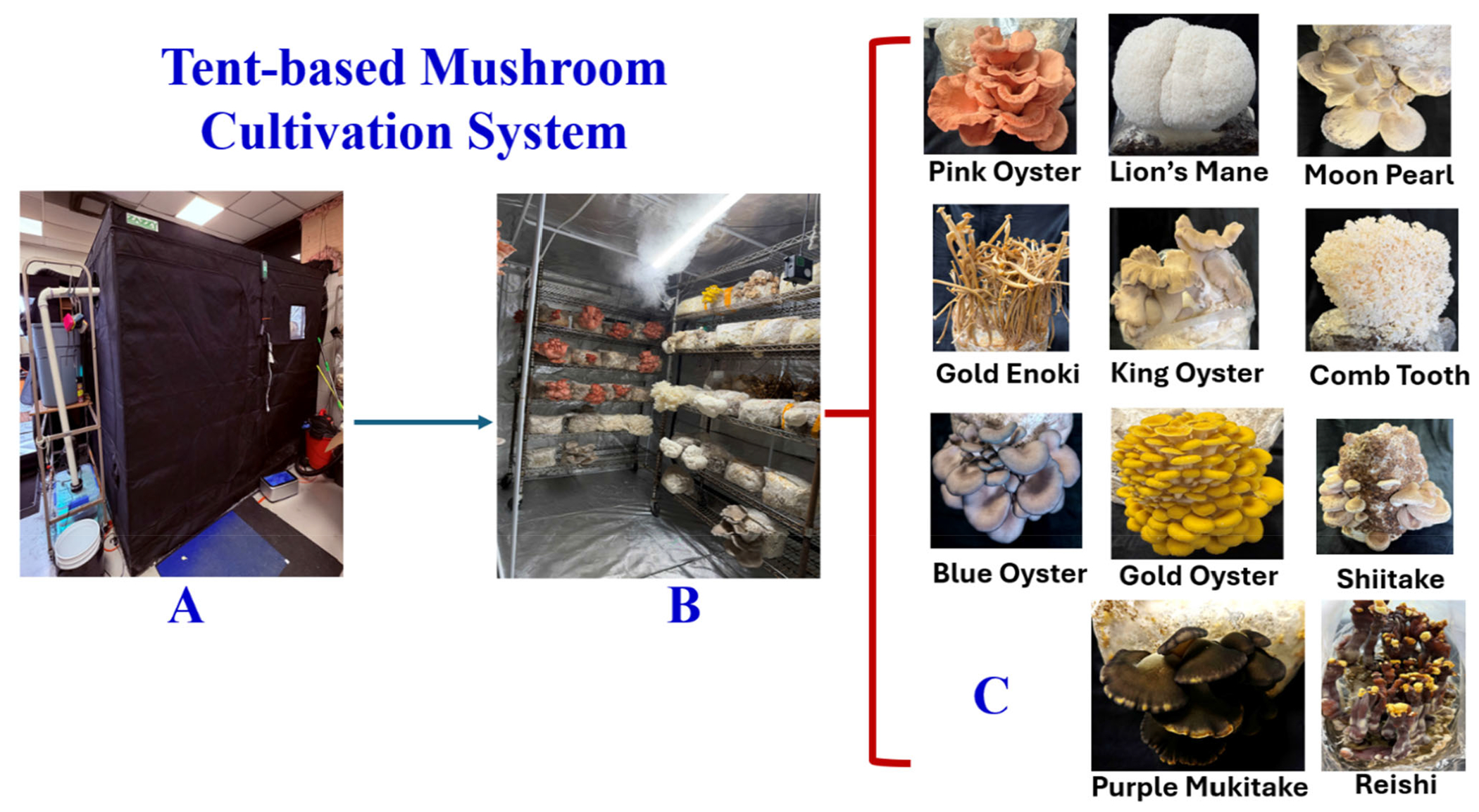
Tent-based mushroom cultivation system for controlled-environment fruiting. (**A**) External view of a portable grow-tent chamber configured as a modular “room-within-a-room” to isolate and regulate cultivation conditions. (**B**) Interior of the fruiting chamber showing multi-tier shelving units containing substrate bags maintained under controlled humidity, ventilation, and LED lighting, including blue-light supplementation for fruiting induction. (**C**) Representative diversity of cultivated mushroom species produced in the tent system, including oyster mushrooms (*Pleurotus* spp.), lion’s mane (*Hericium erinaceus*), and other specialty fungi.

Two complementary cultivation systems were implemented to support different learning objectives: (i) a commercial AI-assisted automated cultivation platform and (ii) a tent-based cultivation chamber (Figures 1 and 2). The automated platform emphasized monitoring, feedback, and standardized fruiting conditions, whereas the tent-based chamber emphasized hands-on environmental control and troubleshooting.

This dual-platform approach allows students to compare standardized, data-centered cultivation with manual environmental control, while linking fungal biology with CEA and experimental design.

### 2.4. Cultivation Systems and Implementation

An AI-assisted mushroom cultivation system, provided as an in-kind educational gift from Sproushi Company (Cuijk, The Netherlands), was used to provide a controlled and automated growth environment (Figure 1). In this manuscript, “AI-assisted” refers to the commercial system’s sensor/camera monitoring, app-based feedback, and software-assisted environmental regulation. “Automated” refers to system functions that operate without direct manual adjustment, such as regulation of selected fruiting conditions. These terms are not used interchangeably. Based on the manufacturer ’s handbook and product information, the system includes a built-in camera, environmental sensors, mobile-app access, and software-assisted regulation of key fruiting parameters, including humidity, light, and airflow. The manufacturer describes the system as using visual monitoring and growth-stage information to adjust environmental conditions and provide user feedback during cultivation. In this study, the system was used as a commercial AI-assisted cultivation platform rather than as a student-programmed AI tool. Students accessed the system through a mobile application that provided real-time environmental data and system feedback, supporting data literacy and analysis of environmental growth relationships.

In parallel, a tent-based cultivation chamber was constructed as a scalable, hands-on system (Figure 2). The chamber consisted of a portable grow tent equipped with a humidifier, ventilation fan, and LED lighting. Environmental conditions were manually adjusted according to developmental stage, with incubation under low-light conditions followed by fruiting under increased humidity, light exposure, and fresh-air exchange.

Laboratory activities included inoculation, environmental monitoring, growth observation, phenotyping, harvest, and post-harvest analysis. Students collected quantitative and qualitative observations throughout the cultivation process and used these data to interpret growth patterns and environmental responses. The platform supported multiple instructional levels, from guided observation in introductory activities to hypothesis-driven projects in advanced courses, and was also adapted for K–12 outreach.

### 2.5. Educational Integration and System Comparison

By integrating AI-assisted and tent-based systems within a single laboratory environment, the platform provides complementary learning experiences that reflect both modern controlled-environment agriculture and traditional cultivation approaches. This integration was intended to support technical skills, conceptual understanding, data interpretation, and student engagement while maintaining accessibility and scalability for resource-limited educational settings (Table 1).

### 2.6. Student Assessment and Survey Analysis

A pre- and post-course survey was administered to evaluate student learning perceptions associated with the dual-platform mushroom cultivation system. The survey consisted of 11 items designed to assess three domains: (i) perceived conceptual understanding of fungal biology, (ii) technical skills and confidence in mushroom cultivation, and (iii) student engagement and interest in STEM learning. The survey was developed specifically for this educational implementation through instructor/coauthor discussion and was not adapted from a previously validated instrument. No formal validation or reliability testing was performed. Therefore, the results should be interpreted as preliminary self-reported student perceptions rather than validated measures of learning outcomes.

Each item was rated on a 10-point Likert scale (1 = strongly disagree, 10 = strongly agree). The survey sample included 30 students who participated in the related mushroom cultivation activities during the implementation period and completed matched pre- and post-course surveys. Only matched responses were included in the analysis. Responses were collected anonymously and paired for pre/post analysis.

Because the survey used Likert-type self-assessment items, analyses were treated as exploratory. The full list of survey questions is provided in Supplementary Table S1. Full statistics are provided in Supplementary Table S2.

### 2.7. Ethics, Consent, Data Privacy, and Food Safety

The University of Pikeville Institutional Review Board reviewed the student survey component and determined it to be exempt. Survey participation was voluntary, responses were collected anonymously, and results were analyzed and reported only in aggregate. No identifiable survey data are reported in this manuscript. For K–12 outreach demonstrations, no survey data from minors were included in the analysis. Permission was obtained for publication of identifiable images shown in the manuscript. Food-tasting activities used only culinary mushroom species grown in the University of Pikeville mushroom facility. Species identity was verified before use by the course instructors/faculty mycologist, and foods were prepared using standard food-handling practices. Participants were informed that the activity involved edible mushrooms, and allergies or dietary restrictions were considered. The tasting activity was used only as an engagement activity and not as an objective learning assessment.

## 3. Results and Discussion

### 3.1. Enhancing Student Engagement Through Mushroom Cultivation

Student engagement is important in experiential STEM education. In this study, mushroom cultivation served as an accessible entry point because the organisms were familiar, visually dynamic, and capable of rapid development under classroom conditions. Many students were already familiar with edible mushrooms, which helped connect course content with real-world applications.

Unlike common microbiology models, such as bacteria or yeast, macrofungi exhibit visible developmental stages, including mycelial expansion, primordia formation, and fruiting body development. For several cultivated species, these stages can be observed within a single academic term, supporting sustained engagement and conceptual understanding.

**Figure 3.**
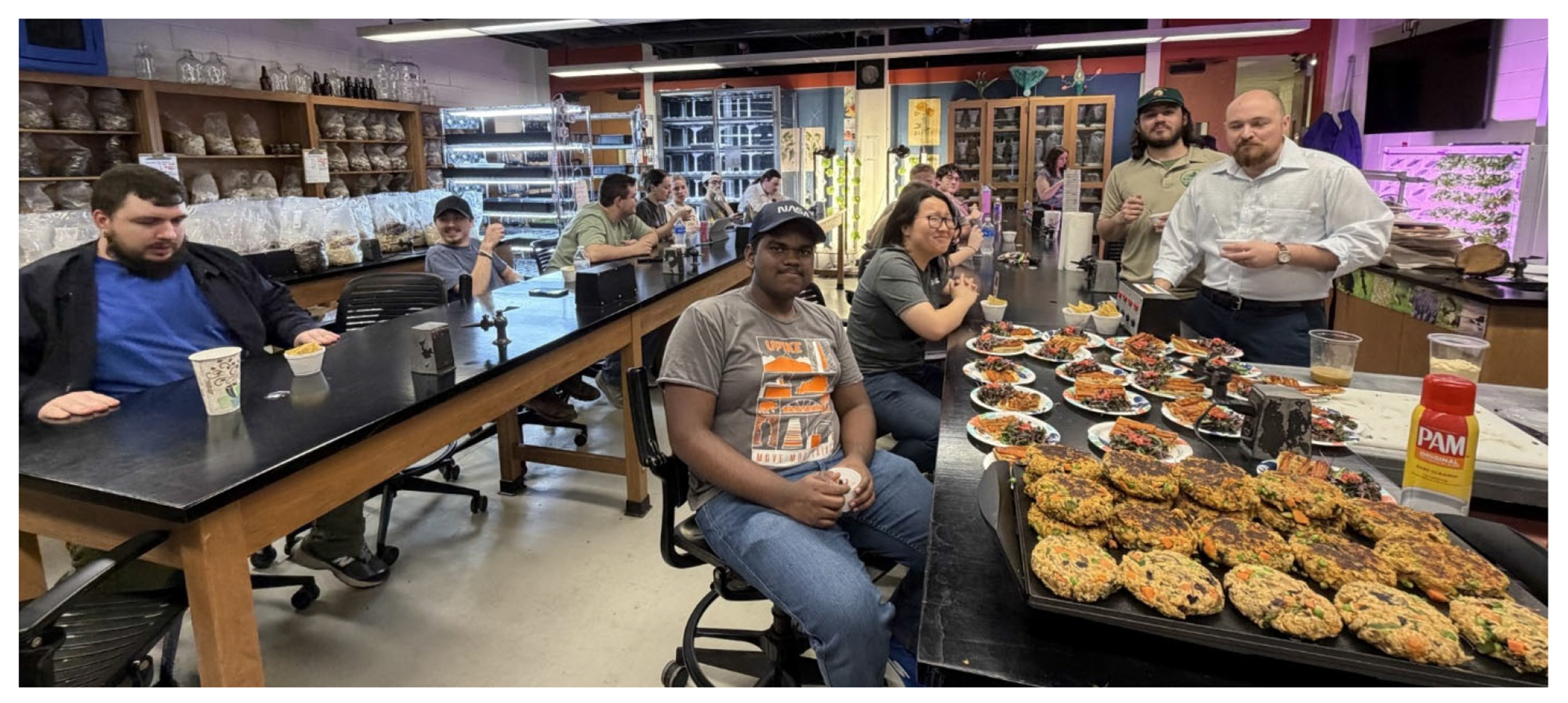
Post-harvest engagement activity associated with the mushroom cultivation platform. Students, faculty, and staff participated in a mushroom-based food tasting session following cultivation and harvest. The activity helped connect fungal biology with food systems, sustainability and real-world applications, and supported student engagement; however, it was not used as an objective measure of learning outcomes.

Post-harvest outcome activities, including preparation and tasting of mushroom-based foods produced through the cultivation system, further connected fungal biology with food systems, sustainability, and everyday life (Figure 3).

### 3.2. Supporting Student Research Interest and Emerging CURE-Based Learning Activities

The dual-platform cultivation system was integrated into BIO 342—Mushrooms & Molds, an upper-level undergraduate course at the University of Pikeville (UPIKE) (approximately 20 students per offering), and into related outreach and laboratory activities. In this initial implementation, the AI-assisted system and tent-based chamber were used to introduce students to fungal biology, controlled-environment agriculture, and data-centered observation. Students engaged in activities spanning the full fungal life cycle, including inoculation, environmental monitoring, growth observation, phenotyping, and post-harvest analysis (Figures 1 and 2).

The cultivation process followed sequential developmental stages: tissue isolation (1–2 days), mycelial growth (3–7 days), grain spawn production (7–21 days), substrate colonization (7–21 days), and fruiting and harvest (3–14 days). This timeline allowed complete growth cycles within approximately 20–40 days for oyster mushrooms and 35–70 days for lion’s mane, enabling implementation within a single academic semester while providing a framework for progressive skill development.

Early stages emphasized aseptic technique and microbial handling, intermediate stages supported experimental design and hypothesis-driven investigation, and final stages enabled quantitative data collection and analysis, such as time to pinning, biomass, and morphology. These activities helped build student interest and provided a foundation for future CURE-based projects involving formal hypotheses, experimental designs, student-generated datasets, and assessment rubrics. All mushroom images shown are authentic images from our cultivation activities. Because formal project-level rubrics and complete student-generated datasets were not collected for all activities during this initial implementation, CURE-related claims are presented cautiously as emerging course-based research development rather than fully assessed CURE outcomes.

### 3.3. Assessment of Student Perceptions and Self-Reported Learning

**Figure 4.**
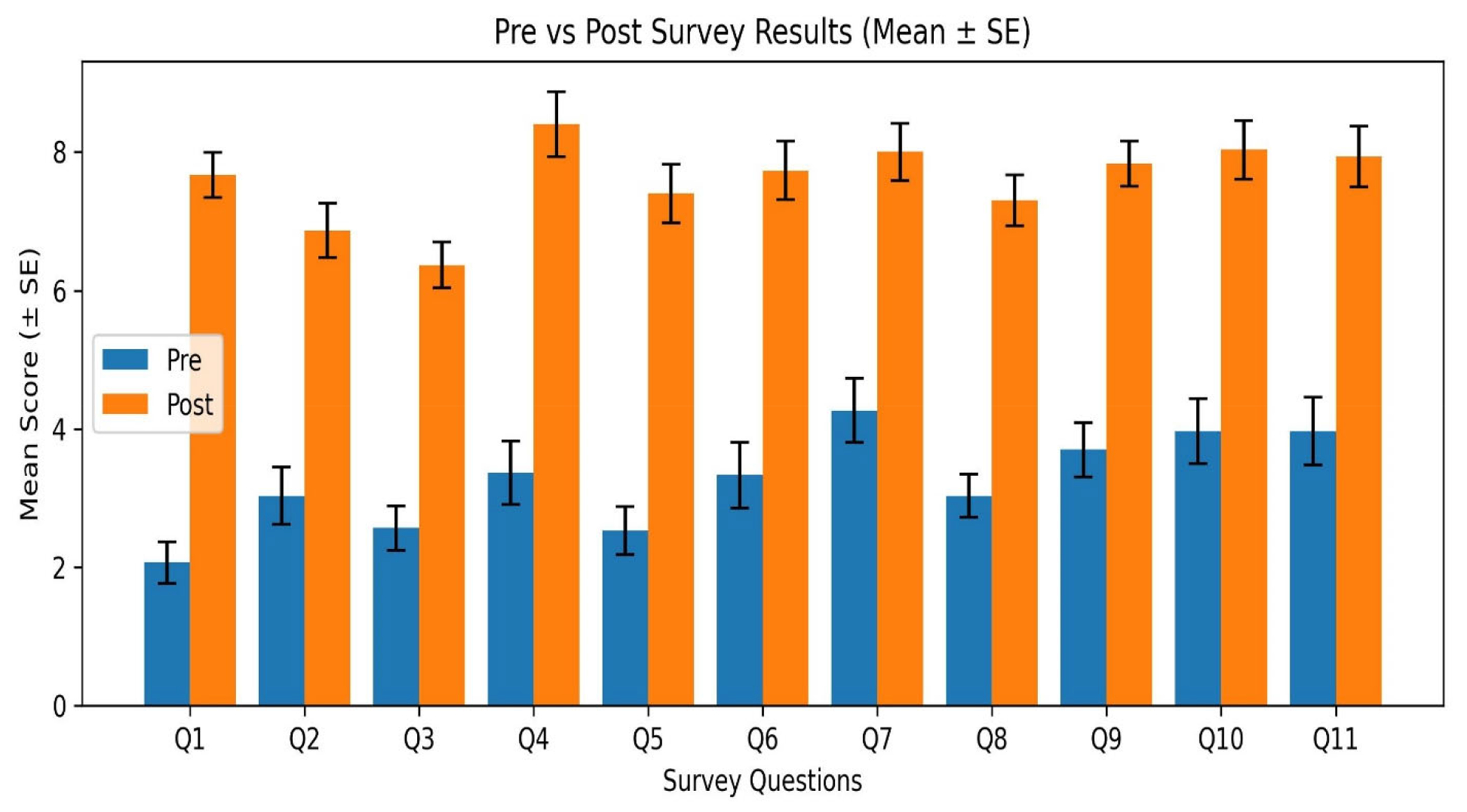
Pre- and post-course survey assessment of student self-reported perceptions, confidence, and engagement in fungal biology and mushroom cultivation (*n* = 30). Bars represent mean selfassessment scores for each survey item on a 10-point Likert scale. Error bars indicate the standard error of the mean (mean ± SE). Scores increased across all items following participation in the dual-platform cultivation system. Exploratory paired *t*-test analysis showed statistically significant differences for all comparisons (*p* < 0.001).

Pre- and post-course survey results showed increases in students’ self-reported perceived understanding and confidence across all assessed categories (Figure 4). Mean self-assessment scores increased consistently from pre-course levels of approximately 2–4 on a 10-point scale to post-course levels of approximately 6–8.

Exploratory paired *t*-test analysis showed that post-course self-assessment scores were significantly higher than pre-course scores across all survey items (*n* = 30, *p* < 0.001 for all comparisons). Because 11 paired comparisons were performed, these analyses should be interpreted cautiously. Supplementary Table S2 provides the full statistical summary, including descriptive statistics, confidence intervals, exact *p*-values, Holm-adjusted *p*-values, effect sizes, Shapiro–Wilk tests of paired differences, and Wilcoxon signed-rank test results. Overall, the findings indicate positive changes in student perceptions, cultivation confidence, and engagement following participation in the dual-platform system. However, they should be interpreted as self-reported outcomes rather than objective evidence of learning gains.

### 3.4. Educational Value of AI-Assisted and Tent-Based Systems

The integration of AI-assisted and tent-based cultivation systems provided complementary instructional functions. The AI-assisted system offered a controlled and reproducible environment for environmental monitoring (Aijaz et al., 2025), app-based feedback, data interpretation, and discussion of environmental-growth relationships.

In contrast, the tent-based system emphasized hands-on manipulation, manual adjustment of cultivation conditions, troubleshooting and system-level understanding. Students directly adjusted humidity, airflow, light exposure, and other environmental parameters, gaining practical experience with cultivation system design and management.

Together, these systems form a dual-platform framework that accommodates diverse instructional levels, from guided laboratory exercises to independent research projects, while integrating concepts in CEA (Boyd et al., 2025) and digital biology (Sangur et al., 2025) The two systems were used within the same instructional implementation but served different learning purposes. Comparative learning data between the two platforms were not collected; therefore, these differences are presented as observed instructional functions rather than evidence that one system produced stronger educational outcomes than the other.

### 3.5. Institutional Impact and STEM Outreach

At a primarily undergraduate institution such as UPIKE, where access to controlled-environment facilities is limited, the dual-platform approach provides an accessible way to integrate hands-on fungal biology into the curriculum, particularly through the lower-cost tent-based component (Supplementary Table S3). Its implementation expanded experiential learning opportunities and strengthened research integration within undergraduate education.

The platform also demonstrated outreach potential. More than 600 K–12 students from the Pikeville, Kentucky region participated in laboratory visits and demonstrations, increasing awareness of fungal biology and controlled-environment agriculture. Several local schools are planning to adopt similar systems, highlighting the scalability of this approach across educational levels.

This model is particularly relevant in rural regions such as Central Appalachia, where access to advanced laboratory infrastructure is limited, but opportunities for community-based STEM engagement are significant.

### 3.6. Limitations and Future Directions

This study is based primarily on instructional observations, student outputs, and self-reported pre- and post-course survey responses rather than objective learning assessments or validated educational instruments. The study did not include a control or comparison group, and the observed changes cannot be causally attributed to the dual-platform intervention. Therefore, the survey findings should be interpreted as preliminary evidence of student perceptions, confidence, and engagement, rather than direct evidence of objective learning gains. Students’ prior experience with fungi or mushroom cultivation was not formally assessed, and the results may also have been influenced by novelty effects or instructor effects.

In addition, the survey was developed specifically for this course-based educational implementation and was not formally validated. Formal project rubrics, complete student-generated datasets, and systematic assessment of hypothesis-driven CURE projects were not collected during this initial implementation. Future work will incorporate structured and performance-based assessment tools, including graded assignments, lab reports, rubrics, conceptual questions, student-generated environmental-growth data analyses, and more formal CURE project assessment.

Variability in the tent-based system may affect reproducibility in student-managed experiments; however, this variability reflects authentic research conditions and contributes to experiential learning.

Future directions include expanding the platform to additional fungal species, integrating molecular and biotechnological analyses, and developing standardized instructional modules to support broader adoption.

## 4. Conclusions

The dual-platform mushroom cultivation system integrating AI-assisted environmental monitoring and controlled-environment agriculture provides an accessible approach for experiential STEM education. By enabling semester-length observation of fungal development within existing laboratory spaces, this platform addresses key limitations of traditional instruction.

At UPIKE, integration of the platform into coursework supported hands-on learning, environmental observation, data interpretation, and student engagement. The AI-assisted and tent-based systems provided complementary instructional functions across undergraduate learning and outreach contexts.

Beyond undergraduate education, the platform demonstrated outreach potential for K–12 audiences, particularly in rural regions such as Central Appalachia. Its adaptability, especially through the lower-cost tent-based system, makes it a practical model for integrating fungal biology and controlled-environment agriculture into STEM education. Future studies using objective assessments, validated instruments, and comparison groups will be needed to evaluate learning outcomes more directly.

## Supplementary Material

Supplementary Material

The following supporting information can be downloaded at:https://www.mdpi.com/article/10.3390/educsci16071010/s1, Supplementary Table S1: Pre- and post-course survey instrument used to assess student learning outcomes in fungal biology and mushroom cultivation. Supplementary Table S2: Descriptive and inferential statistics for pre- and post-course student self-assessment survey items. Supplementary Table S3: Practical implementation guide for implementing the dual-platform mushroom cultivation system.

## Figures and Tables

**Table 1.** Comparison of AI-assisted and tent-based mushroom cultivation systems used in this study.

Feature	AI-Assisted System (e.g., Sproushi)	Tent-Based System
Setup complexity	Low (plug-and-play)	Moderate (assembly required)
Cost	Higher per unit	Lower; scalable
Capacity	Limited	High (multi-shelf capacity)
Environmental control	Automated (AI-driven)	Manual or semi-automated
Data monitoring	Real-time (mobile application)	Sensor-based (controller dependent)
Student interaction	Monitoring and data analysis	Direct manipulation and control
Experimental flexibility	Moderate	High
Reproducibility	High (standardized conditions)	Variable (user-dependent)
Educational emphasis	Environmental monitoring; data literacy	Hands-on skills; system design
Typical use	Guided monitoring activities	Hands-on laboratories and emerging CURE projects

## Data Availability

The original contributions presented in this study are included in the article/Supplementary Material. Further inquiries can be directed to the corresponding authors.
