# Supplementary material for: Dual-Platform Mushroom Cultivation for STEM Education: AI-Assisted Environmental Monitoring and Student Perceptions
Source: Educ Sci (Basel). Author manuscript; Available in PMC 2026 Jul 21. (PMC13384478; doi:10.3390/educsci16071010)
Supplement: Supplementary Material [file NIHMS2191557-supplement-Supplementary_Material.zip › Supplementary Table S1.pdf]

Supplementary Table S1. Pre- and post-course survey instrument used to assess student learning outcomes in fungal biology and mushroom cultivation.

No.	Survey Statement	Pre (1–10)	Post (1–10)
1	I can grow my own mushroom.		
2	I understand the basic biology of fungi.		
3	I understand the major stages of fungal development.		
4	I understand the environmental conditions required for mushroom cultivation.		
5	I can explain how humidity, airflow, light, and substrate affect mushroom growth.		
6	I feel confident participating in mushroom cultivation activities.		
7	I feel confident collecting and interpreting growth data.		
8	I feel confident designing or modifying a simple cultivation experiment.		
9	Mushroom cultivation helps me connect biology to real-world applications.		
10	This activity increases my engagement in STEM learning.		
11	I am interested in learning more about fungal biology after this experience.		
