# Supplementary material for: Dual-Platform Mushroom Cultivation for STEM Education: AI-Assisted Environmental Monitoring and Student Perceptions
Source: Educ Sci (Basel). Author manuscript; Available in PMC 2026 Jul 21. (PMC13384478; doi:10.3390/educsci16071010)
Supplement: Supplementary Material [file NIHMS2191557-supplement-Supplementary_Material.zip › Supplementary Table S2.pdf]

Supplementary Table S2. Descriptive and inferential statistics for pre- and post-course student self-assessment survey items.

Data are paired pre/post Likert-type self-assessment scores from 30 students. Mean difference = post-course score minus pre-course score. Exact paired t-test p values, Holm-adjusted p values, Cohen's dz, Shapiro-Wilk p values for paired differences, and Wilcoxon signed-rank test p values are reported. Analyses should be interpreted as exploratory and as self-reported student perceptions rather than objective evidence of learning gains.

No.	Survey statement	n	Pre Mean	Pre SD
1	I can grow my own mushroom.	30	2.07	1.72
2	I understand the basic biology of fungi.	30	3.03	2.36
3	I understand the major stages of fungal development.	30	2.57	1.81
4	I understand the environmental conditions required for mushroom cultivation.	30	3.37	2.61
5	I can explain how humidity, airflow, light, and substrate affect mushroom growth.	30	2.53	1.96
6	I feel confident participating in mushroom cultivation activities.	30	3.33	2.68
7	I feel confident collecting and interpreting growth data.	30	4.27	2.61
8	I feel confident designing or modifying a simple cultivation experiment.	30	3.03	1.77
9	Mushroom cultivation helps me connect biology to real-world applications.	30	3.70	2.23
10	This activity increases my engagement in STEM learning.	30	3.97	2.63
11	I am interested in learning more about fungal biology after this experience.	30	3.97	2.79

Supplementary Table S2. Descriptive and inferential statistics for pre- and post-course student self-assessment survey items.

Data are paired pre/post Likert-type self-assessment scores from 30 students. Mean difference = post-course score minus pre-course score. Exact paired t-test p values, Holm-adjusted p values, Cohen's dz, Shapiro-Wilk p values for paired differences, and Wilcoxon signed-rank test p values are reported. Analyses should be interpreted as exploratory and as self-reported student perceptions rather than objective evidence of learning gains.

No.	Survey statement	Post Mean	Post SD	Mean Difference (Post-Pre)
1	I can grow my own mushroom.	7.67	1.83	5.60
2	I understand the basic biology of fungi.	6.87	2.22	3.83
3	I understand the major stages of fungal development.	6.37	1.87	3.80
4	I understand the environmental conditions required for mushroom cultivation.	8.40	2.63	5.03
5	I can explain how humidity, airflow, light, and substrate affect mushroom growth.	7.40	2.40	4.87
6	I feel confident participating in mushroom cultivation activities.	7.73	2.39	4.40
7	I feel confident collecting and interpreting growth data.	8.00	2.33	3.73
8	I feel confident designing or modifying a simple cultivation experiment.	7.30	2.07	4.27
9	Mushroom cultivation helps me connect biology to real-world applications.	7.83	1.86	4.13
10	This activity increases my engagement in STEM learning.	8.03	2.40	4.07
11	I am interested in learning more about fungal biology after this experience.	7.93	2.49	3.97

Supplementary Table S2. Descriptive and inferential statistics for pre- and post-course student self-assessment survey items.

Data are paired pre/post Likert-type self-assessment scores from 30 students. Mean difference = post-course score minus pre-course score. Exact paired t-test p values, Holm-adjusted p values, Cohen's dz, Shapiro-Wilk p values for paired differences, and Wilcoxon signed-rank test p values are reported. Analyses should be interpreted as exploratory and as self-reported student perceptions rather than objective evidence of learning gains.

No.	Survey statement	Mean Difference	95% CI Lower	95% CI Upper
1	I can grow my own mushroom.	5.60	4.69	6.51
2	I understand the basic biology of fungi.	3.83	3.01	4.66
3	I understand the major stages of fungal development.	3.80	3.12	4.48
4	I understand the environmental conditions required for mushroom cultivation.	5.03	3.80	6.26
5	I can explain how humidity, airflow, light, and substrate affect mushroom growth.	4.87	3.60	6.13
6	I feel confident participating in mushroom cultivation activities.	4.40	3.37	5.43
7	I feel confident collecting and interpreting growth data.	3.73	2.78	4.69
8	I feel confident designing or modifying a simple cultivation experiment.	4.27	3.37	5.16
9	Mushroom cultivation helps me connect biology to real-world applications.	4.13	3.25	5.02
10	This activity increases my engagement in STEM learning.	4.07	3.09	5.05
11	I am interested in learning more about fungal biology after this experience.	3.97	2.98	4.95

Supplementary Table S2. Descriptive and inferential statistics for pre- and post-course student self-assessment survey items.

Data are paired pre/post Likert-type self-assessment scores from 30 students. Mean difference = post-course score minus pre-course score. Exact paired t-test p values, Holm-adjusted p values, Cohen's dz, Shapiro-Wilk p values for paired differences, and Wilcoxon signed-rank test p values are reported. Analyses should be interpreted as exploratory and as self-reported student perceptions rather than objective evidence of learning gains.

No.	Survey statement	t	Exact p value	Holm-adjusted p
1	I can grow my own mushroom.	12.62	2.62E-13	2.88E-12
2	I understand the basic biology of fungi.	9.48	2.17E-10	1.54E-09
3	I understand the major stages of fungal development.	11.51	2.47E-12	2.47E-11
4	I understand the environmental conditions required for mushroom cultivation.	8.37	3.21E-09	1.28E-08
5	I can explain how humidity, airflow, light, and substrate affect mushroom growth.	7.86	1.14E-08	1.64E-08
6	I feel confident participating in mushroom cultivation activities.	8.73	1.32E-09	7.92E-09
7	I feel confident collecting and interpreting growth data.	7.99	8.19E-09	1.64E-08
8	I feel confident designing or modifying a simple cultivation experiment.	9.77	1.11E-10	1.00E-09
9	Mushroom cultivation helps me connect biology to real-world applications.	9.54	1.92E-10	1.54E-09
10	This activity increases my engagement in STEM learning.	8.49	2.39E-09	1.19E-08
11	I am interested in learning more about fungal biology after this experience.	8.21	4.69E-09	1.41E-08

Supplementary Table S2. Descriptive and inferential statistics for pre- and post-course student self-assessment survey items.

Data are paired pre/post Likert-type self-assessment scores from 30 students. Mean difference = post-course score minus pre-course score. Exact paired t-test p values, Holm-adjusted p values, Cohen's dz, Shapiro-Wilk p values for paired differences, and Wilcoxon signed-rank test p values are reported. Analyses should be interpreted as exploratory and as self-reported student perceptions rather than objective evidence of learning gains.

No.	Survey statement	Cohen's dz	Shapiro-Wilk p	Wilcoxon signed-rank p
1	I can grow my own mushroom.	2.30	3.12E-02	1.87E-06
2	I understand the basic biology of fungi.	1.73	3.87E-01	3.44E-06
3	I understand the major stages of fungal development.	2.10	1.97E-01	3.40E-06
4	I understand the environmental conditions required for mushroom cultivation.	1.53	5.00E-02	6.30E-06
5	I can explain how humidity, airflow, light, and substrate affect mushroom growth.	1.44	5.51E-02	1.01E-05
6	I feel confident participating in mushroom cultivation activities.	1.59	1.06E-01	5.22E-06
7	I feel confident collecting and interpreting growth data.	1.46	2.14E-01	7.90E-06
8	I feel confident designing or modifying a simple cultivation experiment.	1.78	2.77E-01	4.05E-06
9	Mushroom cultivation helps me connect biology to real-world applications.	1.74	5.27E-01	3.60E-06
10	This activity increases my engagement in STEM learning.	1.55	6.60E-02	5.25E-06
11	I am interested in learning more about fungal biology after this experience.	1.50	7.54E-02	7.58E-06

Supplementary Table S2. Descriptive and inferential statistics for pre- and post-course student self-assessment survey items.

Data are paired pre/post Likert-type self-assessment scores from 30 students. Mean difference = post-course score minus pre-course score. Exact paired t-test p values, Holm-adjusted p values, Cohen's dz, Shapiro-Wilk p values for paired differences, and Wilcoxon signed-rank test p values are reported. Analyses should be interpreted as exploratory and as self-reported student perceptions rather than objective evidence of learning gains.

No.	Survey statement	Interpretation
1	I can grow my own mushroom.	Exploratory; self-reported improvement
2	I understand the basic biology of fungi.	Exploratory; self-reported improvement
3	I understand the major stages of fungal development.	Exploratory; self-reported improvement
4	I understand the environmental conditions required for mushroom cultivation.	Exploratory; self-reported improvement
5	I can explain how humidity, airflow, light, and substrate affect mushroom growth.	Exploratory; self-reported improvement
6	I feel confident participating in mushroom cultivation activities.	Exploratory; self-reported improvement
7	I feel confident collecting and interpreting growth data.	Exploratory; self-reported improvement
8	I feel confident designing or modifying a simple cultivation experiment.	Exploratory; self-reported improvement
9	Mushroom cultivation helps me connect biology to real-world applications.	Exploratory; self-reported improvement
10	This activity increases my engagement in STEM learning.	Exploratory; self-reported improvement
11	I am interested in learning more about fungal biology after this experience.	Exploratory; self-reported improvement
