# Supplementary material for: Dual-Platform Mushroom Cultivation for STEM Education: AI-Assisted Environmental Monitoring and Student Perceptions
Source: Educ Sci (Basel). Author manuscript; Available in PMC 2026 Jul 21. (PMC13384478; doi:10.3390/educsci16071010)

Practical implementation guide for implementing the dual-platform mushroom cultivation system.

Category	Required Components	Min Cost	Max Cost
Tent enclosure	4×4 grow tent, waterproof floor tray, zipper access, intake/exhaust ports	$160	$250
Shelving / crop support	1–2 wire shelving racks, plastic trays under blocks, shelf liners	$75	$250
Humidification	Ultrasonic humidifier, fogger, or bucket fogger; ideally external reservoir with ducted fog	$70	$200
Humidity control	Humidistat/controller, RH probe, plug outlet control	$60	$160
Fresh air exchange / exhaust	4-inch inline fan, ducting, clamps, exterior vent or room exhaust path	$120	$250
Internal air circulation	1–2 clip fans inside tent	$45	$120
Lighting	Low-intensity LED bar or blue/full-spectrum supplemental lights on timer	$40	$120
Sensors / monitoring	Temp/RH sensor; optional CO_2 sensor; optional data logger	$25	$200
Software / app	AC Infinity app, Inkbird app, or equivalent for Wi-Fi/Bluetooth monitoring; 2.4 GHz Wi-Fi may be required for Wi-Fi controllers	$0	$20
Electrical / water safety	GFCI-protected outlet, surge strip mounted above floor, drip loops, cord management	$40	$100
Cleaning / sanitation supplies	Spray bottles, peroxide or approved sanitizer, alcohol wipes for tools, paper towels, trash bags, dedicated harvest bins	$50	$150
PPE	Nitrile gloves, masks/N95s for heavy spore exposure or cleanup, eye protection for cleaning	$30	$100
Consumables / crop inputs	Fruiting blocks or grow bags, substrate, spawn, labels, harvest containers	$75	$300
Replacement parts reserve	Spare humidifier disc, tubing, duct clamps, fan, RH probe, filters, extra tray	$100	$250

Practical implementation guide for implementing the dual-platform mushroom cultivation system.

Category	Maintenance / Replacement Needs	Midpoint	Required?
Tent enclosure	Wipe down after each crop; inspect zippers, seams, and floor tray; replace tent every 3–5 years if heavily used	$205	Yes
Shelving / crop support	Clean shelves between cycles; watch for rust; replace trays when cracked	$163	Yes
Humidification	Use clean water; descale weekly; replace ultrasonic discs, wicks, or reservoir parts as needed	$135	Yes
Humidity control	Calibrate/check RH sensor monthly; keep probe out of direct fog stream	$110	Yes
Fresh air exchange / exhaust	Clean fan blades/ducting; inspect condensation; replace ducting if moldy	$185	Yes
Internal air circulation	Wipe fan guards; avoid blowing directly on blocks; replace failed fans	$83	Yes
Lighting	Wipe condensation; replace failed strips; keep cords off wet floor	$80	Yes
Sensors / monitoring	Sensor check monthly; keep backup thermometer/hygrometer	$113	Recommended
Software / app	Maintain login, Wi-Fi access, and shared device permissions; export data weekly for student projects	$10	Recommended
Electrical / water safety	Monthly inspection; keep all plugs off the floor; remove damaged cords immediately	$70	Yes
Cleaning / sanitation supplies	Clean between crops; remove contaminated blocks immediately; keep written cleaning checklist	$100	Yes
PPE	Restock gloves/masks; require PPE during contamination cleanup	$65	Yes
Consumables / crop inputs	Track strain, date, substrate, contamination, yield; replace contaminated blocks	$188	Yes
Replacement parts reserve	Keep one spare humidifier or fogger per 3–5 tents	$175	Recommended

Practical implementation guide for implementing the dual-platform mushroom cultivation system.

Category	Replacement Parts	Notes
Tent enclosure	Zipper pulls, floor tray, patch tape	Assumes a 48×48×80 in grow tent.
Shelving / crop support	Plastic trays, shelf liners, shelf clips	Avoid raw wood in humid chambers.
Humidification	Ultrasonic discs, tubing, reservoir cap/gasket	External humidifiers are easier to service and keep electronics out of the tent.
Humidity control	RH probe, controller outlet	Essential for repeatable chamber conditions.
Fresh air exchange / exhaust	Ducting, clamps, fan, optional pre-filter	Do not exhaust spores into a small classroom or office.
Internal air circulation	Clip fans, USB adapters	Circulation reduces stagnant pockets but can dry fruits if aimed directly at blocks.
Lighting	LED strip/bar, timer	Fruiting usually needs low light, not high-intensity plant grow lighting.
Sensors / monitoring	Sensor batteries, spare thermometer/hygrometer, CO_2 sensor if used	CO_2 is optional but helpful for student research.
Software / app	None; account/app access	May require campus IT approval or a non-campus network.
Electrical / water safety	GFCI adapter/outlet, cord clips, surge strip	Non-negotiable in wet humid environments.
Cleaning / sanitation supplies	Spray bottles, sanitizer, wipes, paper towels, trash bags	Use campus-approved disinfectants and document cleaning.
PPE	Gloves, N95s, eye protection	Required for cleanup and recommended for harvesting heavy-sporing species.
Consumables / crop inputs	Grow bags, substrate, spawn, labels, harvest containers	Recurring per crop cycle; not included in startup total unless starting first cycle.

Replacement parts reserve	Humidifier disc, tubing, clamps, fan, RH probe, tray	Annual reserve; prudent for outreach or semester schedules.

Practical implementation guide for implementing the dual-platform mushroom cultivation system.

Category	Low-Resource Adaptation
Tent enclosure	2×4 tent, mini greenhouse, or clear plastic shelving greenhouse; lower capacity and less stable humidity
Shelving / crop support	Plastic utility shelves or milk crates; avoid raw wood because it traps moisture and contamination
Humidification	Hand misting for demos only; poor for research because RH fluctuates
Humidity control	Manual hygrometer + spray bottle; acceptable for observation labs, not quantitative trials
Fresh air exchange / exhaust	Small computer fan or bathroom-style exhaust fan; weaker CO_2 and spore control
Internal air circulation	Small USB fans; lower airflow but workable for small tents
Lighting	Ambient room light near a window; avoid direct sun and heat spikes
Sensors / monitoring	Manual readings twice daily using cheap digital hygrometer
Software / app	No app; use paper log sheets and manual set points
Electrical / water safety	Use only one humidifier + one fan + one light to reduce electrical load
Cleaning / sanitation supplies	Soap/water + dilute bleach for non-food-contact surfaces; still document cleaning
PPE	Gloves + surgical masks for demos; N95s for cleanup or heavy sporulation
Consumables / crop inputs	Buy ready-to-fruit blocks; avoids sterilization/inoculation infrastructure
Replacement parts reserve	Share replacement parts across multiple units

Startup Cost Summary: Startup minimum excluding crop inputs: $815; Startup maximum excluding crop inputs: $2,170

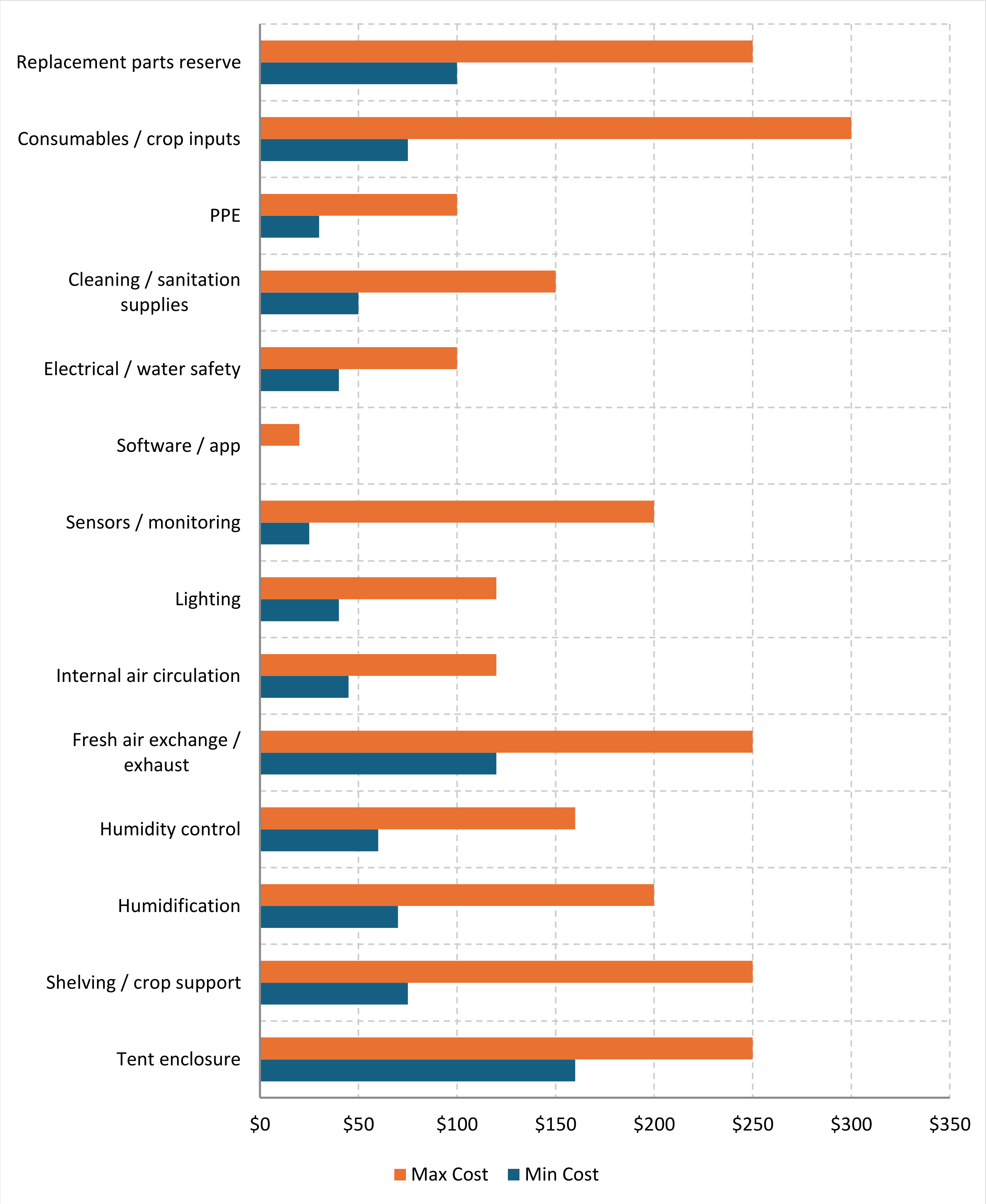

Supplement: Supplementary Material [file NIHMS2191557-supplement-Supplementary_Material.zip › Supplementary Table S3.pdf]
